# Profiling of the Tox21 Chemical Collection for Mitochondrial Function to Identify Compounds that Acutely Decrease Mitochondrial Membrane Potential

**DOI:** 10.1289/ehp.1408642

**Published:** 2014-10-10

**Authors:** Matias S. Attene-Ramos, Ruili Huang, Sam Michael, Kristine L. Witt, Ann Richard, Raymond R. Tice, Anton Simeonov, Christopher P. Austin, Menghang Xia

**Affiliations:** 1 National Center for Advancing Translational Sciences, National Institutes of Health (NIH), Department of Health and Human Services (DHHS), Bethesda, Maryland, USA; 2 Division of the National Toxicology Program, National Institute of Environmental Health Sciences, NIH, DHHS, Research Triangle Park, North Carolina, USA; 3 National Center for Computational Toxicology, Office of Research and Development, U.S. Environmental Protection Agency, Research Triangle Park, North Carolina, USA

## Abstract

Background: Mitochondrial dysfunction has been implicated in the pathogenesis of a variety of disorders including cancer, diabetes, and neurodegenerative and cardiovascular diseases. Understanding whether different environmental chemicals and druglike molecules impact mitochondrial function represents an initial step in predicting exposure-related toxicity and defining a possible role for such compounds in the onset of various diseases.

Objectives: We sought to identify individual chemicals and general structural features associated with changes in mitochondrial membrane potential (MMP).

Methods: We used a multiplexed [two end points in one screen; MMP and adenosine triphosphate (ATP) content] quantitative high throughput screening (qHTS) approach combined with informatics tools to screen the Tox21 library of 10,000 compounds (~ 8,300 unique chemicals) at 15 concentrations each in triplicate to identify chemicals and structural features that are associated with changes in MMP in HepG2 cells.

Results: Approximately 11% of the compounds (913 unique compounds) decreased MMP after 1 hr of treatment without affecting cell viability (ATP content). In addition, 309 compounds decreased MMP over a concentration range that also produced measurable cytotoxicity [half maximal inhibitory concentration (IC_50_) in MMP assay/IC_50_ in viability assay ≤ 3; *p* < 0.05]. More than 11% of the structural clusters that constitute the Tox21 library (76 of 651 clusters) were significantly enriched for compounds that decreased the MMP.

Conclusions: Our multiplexed qHTS approach allowed us to generate a robust and reliable data set to evaluate the ability of thousands of drugs and environmental compounds to decrease MMP. The use of structure-based clustering analysis allowed us to identify molecular features that are likely responsible for the observed activity.

Citation: Attene-Ramos MS, Huang R, Michael S, Witt KL, Richard A, Tice RR, Simeonov A, Austin CP, Xia M. 2015. Profiling of the Tox21 chemical collection for mitochondrial function to identify compounds that acutely decrease mitochondrial membrane potential. Environ Health Perspect 123:49–56; http://dx.doi.org/10.1289/ehp.1408642

## Introduction

Mitochondria are intracellular organelles found in most eukaryotic cells (Bereiter-Hahn and Vöth 1994). In general, these organelles have an outer membrane and an inner membrane characterized by protrusions into the interior matrix. The shape and number of mitochondria vary extensively among different cell types (Bereiter-Hahn and Vöth 1994; Sjostrand 1953). Mitochondria play a central role in maintaining cellular homeostasis, being involved in a variety of critical cellular processes from macromolecular synthesis to the regulation of programed cell death (Dykens et al. 1999). Most of the cell energy is generated as adenosine triphosphate (ATP) within the mitochondria through two complementary processes: the tricarboxylic acid cycle (TCA) and oxidative phosphorylation (Barnes and Weitzman 1986; Dykens et al. 1999; Mitchell 1961). Other metabolic processes, including parts of the urea cycle as well as porphyrin and steroid synthesis and maturation, also occur in the mitochondrial matrix (Gamble and Lehninger 1973; Lambeth et al. 1982; Lill et al. 1999). Mitochondria play a critical role in the maintenance of calcium homeostasis in the cytoplasm. The ability of mitochondria to take up and release calcium ions (Ca^2+^) and to regulate the generation of other intracellular messengers, such as reactive oxygen species (ROS), makes this organelle an essential part of cell signaling (McBride et al. 2006). Mitochondria are also involved in controlling cell fate by regulating cell cycle progression and the initiation of apoptosis (Lemasters et al. 2002; Pacher and Hajnóczky 2001).

Mitochondrial dysfunction (due to either genetic or environmental factors) has been related to many disorders, including cancer, diabetes, and neurodegenerative and cardiovascular diseases (Lesnefsky et al. 2001; Pieczenik and Neustadt 2007). A bioenergetic imbalance, an increase of mitochondria-related oxidative stress, or a deregulation of the intrinsic apoptotic pathway seems to play a role in the onset of many of these diseases at a molecular level (Pieczenik and Neustadt 2007). A similar role for mitochondria-mediated oxidative stress has been proposed in a model for the aging process (Lenaz 1998). Moreover, many clinically approved drugs have been removed from the market as a result of cardiovascular and liver toxicities associated with impaired mitochondrial function (Nadanaciva and Will 2011). Understanding how different chemicals can affect mitochondrial activity constitutes the first step in predicting possible exposure-related toxic effects from such compounds. Following the generation of mitochondrial toxicity profiles, the next challenge is defining a potential role for these chemicals in the onset of various syndromes or diseases linked to mitochondrial dysfunction (Schmidt 2010).

The Tox21 collaboration, which includes the National Toxicology Program (NTP), the U.S. Environmental Protection Agency (EPA) National Center for Computational Toxicology (NCCT), the National Institutes of Health (NIH) Chemical Genomics Center (NCGC; now part of the National Center for Advancing Translational Sciences), and the U.S. Food and Drug Administration (FDA), has goals that include identifying mechanisms of chemically induced biological activity, prioritizing untested chemicals for more extensive toxicological evaluation, and developing predictive models of *in vivo* biological responses (Kavlock et al. 2009; Tice et al. 2013). The ultimate objective of the Tox21 program is to incorporate advances in molecular systems and computational biology into toxicity evaluations in order to study the ever-increasing number of chemicals present in the environment and to accelerate the transition of toxicology into a predictive science focused on mechanism-based biological observations (NTP 2004). To rapidly and efficiently evaluate thousands of compounds across many biologically relevant targets, we implemented an automated robotic system in the Tox21 compound-screening process (Attene-Ramos et al. 2013b). During Tox21 Phase I (proof of principle), many biochemical and cell-based assays were successfully developed and miniaturized in a 1,536-well plate format for screening against the initial Tox21 collection of approximately 3,000 compounds in a quantitative high throughput screening (qHTS) platform (Shukla et al. 2010; Tice et al. 2013). In Phase II (production phase), the Tox21 compound library was expanded to > 10,000 samples (10K), including ~ 8,300 unique chemical entities, selected by each of the Tox21 partners based on public health and toxicological relevance (Attene-Ramos et al. 2013b).

To identify environmental chemicals and drugs that might affect mitochondria dysfunction, we used a qHTS approach to evaluate the compounds from the Tox21 10K compound library that potentially reduce the mitochondrial membrane potential (MMP) in human liver carcinoma (HepG2) cells. Compounds affecting mitochondrial function and structural integrity by different mechanism would likely change the MMP, making this measure a suitable comprehensive first end point for screening. The MMP screen was multiplexed with a viability assay to identify compounds that induced concurrent cytotoxicity, a potential confounder based on the assay format. Using this approach, we identified a group of known and novel compounds with the ability to reduce MMP, and we identified several structural features associated with the observed biological activity.

## Materials and Methods

*Reagents and Tox21 10K compound library*. We purchased human HepG2 (hepatocellular carcinoma) cells from the American Type Culture Collection (ATCC, Manassas, VA); Mitochondrial Membrane Potential Indicator (m-MPI) from Codex Biosolutions (Montgomery Village, MD); and carbonyl cyanide 4-(trifluoromethoxy) phenylhydrazone (FCCP) from Sigma-Aldrich (St. Louis, MO). Intracellular ATP content, an indicator of cell viability, was measured using the CellTiter-Glo® luminescence cell viability assay (Promega, Madison, WI). Detailed compound library information is provided in Supplemental Material, “Compound library.”

*qHTS using a multiplex mitochondrial membrane potential and cell viability assay*. The multiplex assay consisted of two end points, MMP and total intracellular ATP, measured in HepG2 cells in the same assay well after 1 hr of treatment. The assay was optimized using m-MPI (Sakamuru et al. 2012) to measure changes in MMP and CellTiter-Glo® reagent to measure intracellular ATP levels (Crouch et al. 1993). The assay protocol is provided in Supplemental Material, “Quantitative high throughput screening (qHTS) of mitochondrial membrane potential and cell viability multiplex assay” and summarized in Supplemental Material, Table S1. The assay plate map is shown in Supplemental Material, Figure S1.

*qHTS data analysis*. Analysis of compound concentration–response data was performed as previously described (Huang et al. 2011; Inglese et al. 2006; see also Supplemental Material, “qHTS data analysis”). For the present study, we defined “antagonist compounds” as compounds that depolarized the mitochondria (decreased the MMP; showed inhibitory curves), and agonist compounds as those compounds that hyperpolarized the mitochondria (increased the MMP; showed activation curves). Single curve activity outcomes were defined as follows: inactive, class 4; active agonist/antagonist, classes 1.1 and 2.1; agonist/antagonist, classes 1.2 and 2.2; and inconclusive agonist/antagonist, all others. Compound-level activity assignment is described below.

*Activity assignments based on triplicate runs*. As shown in Supplemental Material, Figure S2, each curve class was first converted to a curve rank as previously described by Huang et al. (2011), such that more potent and efficacious compounds with higher-quality curves were assigned a higher rank. Curve ranks should be viewed as a numerical measure of compound activity. Curve ranks from replicate runs of a compound were then averaged. The activity outcome of each compound for each of four readouts [ratio, rhodamine (red), FITC (fluorescein isothiocyanate, green), and cell viability] was assigned based on its average curve rank and reproducibility call as shown in Supplemental Material, Table S2. The final MMP activity outcome of each compound was determined based on its multichannel readout activity as shown in Supplemental Material, Table S3.

*Evaluation of curve class reproducibility*. For each sample, all of the replicate curves were compared pairwise for consistency. For each pair of curves, if one was inactive and the other was active, or if one was agonist and the other one was antagonist, an inconsistency count was added to the class for each of the two curves. This process was repeated for all the samples. The inconsistency counts were tallied for each curve class and divided by the total number of comparisons made to yield the curve reproducibility value.

*Chemical structure cluster analysis*. For the purpose of structure clustering, MMP assay results were aggregated to the unique DSSTox (Distributed Structure-Searchable Toxicity) substance level, with substances identified by GSID (Generic Substance ID) or CASRN (Chemical Abstract Services Registry Number). Approximately 200 unique compounds without structures were excluded from this analysis. Compounds in the structured portion of the unique DSSTox compound library (version 3a; http://www.epa.gov/ncct/dsstox/) were clustered based on structural similarity (512-bit ChemAxon® Chemical Fingerprints, Version 6.2.1; ChemAxon; http://www.chemaxon.com/) using the self-organizing map (SOM) algorithm (Kohonen 2006), yielding 651 clusters. Each cluster was evaluated for its enrichment of active antagonists (compared with the library average) using Fisher’s exact test.

## Results

**Figure 1:**
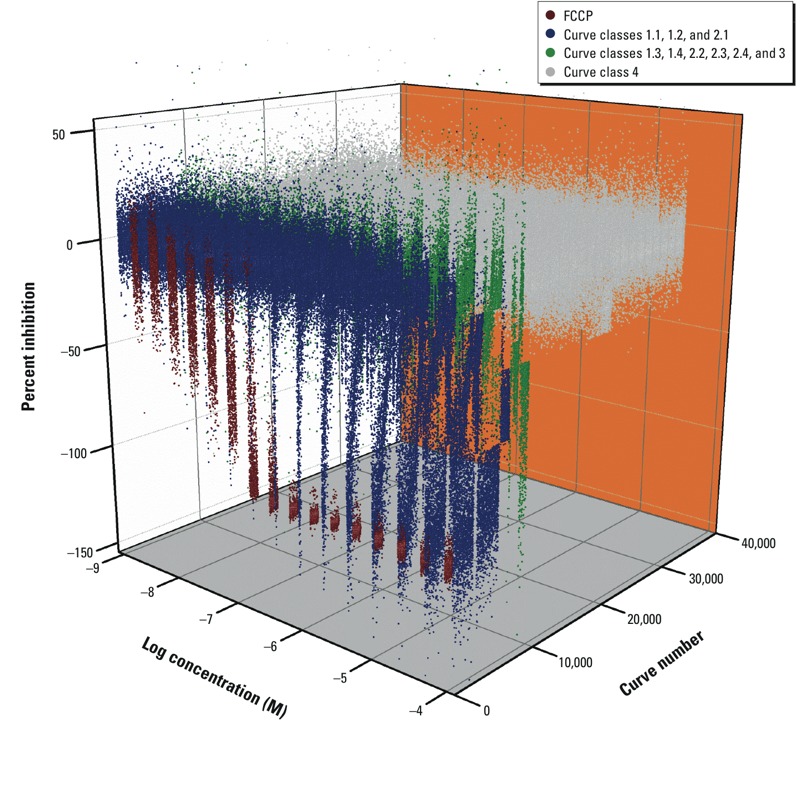
qHTS concentration response data binned into curve classes 1–4. Concentration response [ratio (590 nm/535 nm)] curves for the FCCP control titrations and > 10,000 substances tested, including all the replicates. FCCP is the positive control, and curve class 4 represents the inactive compounds.

*qHTS performance and replicate reproducibility*. As shown in Figure 1 and in Supplemental Material, Figure S3A, the positive control FCCP (red) decreased MMP in a concentration-dependent manner in all 459 plates, with an IC_50_ (half maximal inhibitory concentration) of 0.11 ± 0.07 μM (mean ± SD). For the entire screen, the signal to background (S/B) ratio for the MMP assay was 5.8 ± 2.9 and the *Z*´ factor was 0.64 ± 0.15. The S/B ratio for the cell viability assay was 67.2 ± 16.8 and *Z*´ factor was 0.80 ± 0.10.

As an additional measure of the assay’s technical performance, the reproducibility was evaluated for both the MMP and viability readouts against the 88 compounds present in duplicate in each assay plate (Tox21–88) and against the three copies of the Tox21 10K library with compounds plated in different well locations in each copy (Attene-Ramos et al. 2013b). The reproducibility measures of the Tox21–88 as well as the triplicate assay runs in terms of active match, inactive match, inconclusive, and mismatch rates (see details in Supplemental Material, “Reproducibility call”) and potency differences are listed in Table 1. For both assays, the reproducibility was high, with mismatch rates < 1% of the library and with AC_50_ (concentration at half-maximal activity) fold differences around 1.5 (Table 1).

**Table 1:** Reproducibility for the MMP and cell viability assays.

Assay reproducibility	Active match (%)	Inactive match (%)	Inconclusive (%)	Mismatch (%)	AC_50 _fold change
Tox21–88
MMP	41.88	36.65	19.09	2.39	1.46
Cell viability	8.81	80.63	10.23	0.34	1.41
10K triplicate run
MMP	17.57	67.52	14.33	0.55	1.53
Cell viability	4.78	90.80	4.39	0.03	1.42
For each assay, the reproducibility was calculated for the Tox21–88 compounds (duplicates in each plate) and for the 10K library (three copies) with compounds plated in different well locations.					

**Figure 2:**
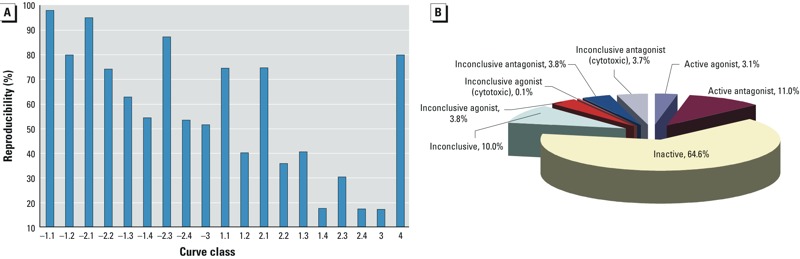
Screening statistics. (*A*) Stability analysis showing the reproducibility of each curve class, calculated using the ratio readout. High-quality curves (i.e., classes 1.1, 1.2, 2.1, 2.2) were more reproducible than other curve types for antagonist activity (inhibitory curve classes); a similar pattern was observed with lower reproducibility for the agonist curves. Class 4 inactive curves were fairly reproducible. (*B*) Distribution of the results (percent of the total library) based on the combined call using the MMP assay (ratio and independent channels), cell viability, and compound autofluorescence assays.

*Evaluation of curve class reproducibility*. The replicate data provided a good opportunity to evaluate the robustness of the process for selecting active compounds based on curve class. The reproducibility of each curve class, calculated using the ratio readout, is shown in Figure 2A. The expectation is that high-quality curves (i.e., class 1.1, 1.2, 2.1, 2.2) are more reproducible and thus compounds in these classes are less likely to be classified as inactive in repeated tests. Figure 2A indicates that this expectation is true for the antagonist curves (negative curve classes), because class –1.1 curves showed the highest reproducibility (98%), followed by class –2.1 curves (95% reproducibility). The other two high-quality curve classes, –1.2 and –2.2, were also more reproducible than the low-quality curves, classes –1.3, –1.4, –2.4, and –3, with the exception of class –2.3 curves, which appeared to be more reproducible than class –1.2 and class –2.2 curves. This may be due to the small sample size (only 50 class –2.3 curves); the average number of other curves was > 1,000 per curve class. A similar pattern was found for the agonist curves (positive curve classes); however, the agonist curve classes showed overall lower reproducibility than their antagonist counterparts. For example, the class 1.1 and 2.1 curves were still the most reproducible (75% reproducibility) among the agonist curves, but they were much less reproducible than the corresponding antagonist curves (–1.1 and –2.1), both of which had > 95% reproducibility. The decreased reproducibility seen for the agonist curves might be the direct consequence of assay design. In this assay, the amount of m-MPI dye that enters the mitochondrial matrix and forms red fluorescence aggregates is proportional to the MMP. We used the uncoupler FCCP to optimize the assay signal window to detect a decrease in red fluorescence (i.e., a decrease in the MMP). An increase in red fluorescence can be detected but the signal window is much smaller. The class 4 (inactive) curves were fairly reproducible (80% reproducibility).

*Identification of environmental compounds and druglike molecules that decrease the MMP*. Decisions on compound activity took into account triplicate runs of not only the ratio (red/green) readout in the MMP assay but also each fluorescence channel individually and the cytotoxicity results (see “Activity assignments based on triplicate runs”; see also Supplemental Material, “Reproducibility call,” Tables S2, S3). Of the 8,312 unique compounds evaluated, 913 (11%) decreased the MMP at 1 hr without apparent cytotoxicity (Figure 2B). Sixty-five percent of the tested compounds (5,366) did not alter the MMP, and 21% (1,778 compounds) produced responses that were considered inconclusive for a variety of reasons. This “inconclusive” group included 309 compounds that were determined to be cytotoxic [IC_50_ in MMP assay/IC_50_ in viability assay ≤ 3, *p* < 0.05]. Conversely, 254 compounds (3%) appeared to increase the MMP. Evaluation of those compounds will be reported elsewhere. The 913 compounds that decreased the MMP without affecting cell viability had IC_50_ values ranging from 9.6 nM to 72 μM, with 79 compounds having an IC_50_ < 1 μM. The protein kinase C (PKC) modulator bryostatin 1 was the most potent compound, with an IC_50_ of 9.6 nM. Other potent compounds included carbocyanine (IC_50_ = 14.1 nM), basic blue 7 (IC_50_ = 18.5 nM), and triethyltin bromide (IC_50_ = 21.7 nM). The 20 most potent compounds are listed in Supplemental Material, Table S4.

**Figure 3:**
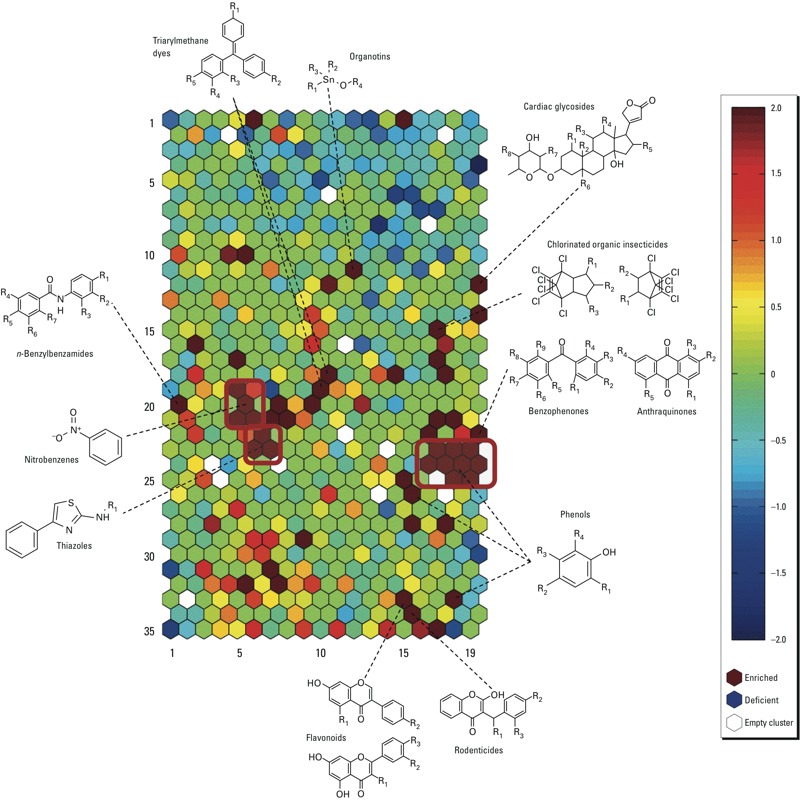
Structure–activity relationship (SAR) of compounds that decreased MMP. All compounds with associated structures present in the library were clustered, based on structural similarity using the self-organizing map (SOM) algorithm (Kohonen 2006). Each cluster was then evaluated for enrichment for active antagonists (compared with the library average) using Fisher’s exact test. Enriched clusters are shown in red and deficient clusters in blue; scale values represent the log *p*-value for each cluster. Representative scaffolds are shown for some of the more enriched clusters. The red boxes define regions of molecules that share a common substructure.

**Figure 4:**
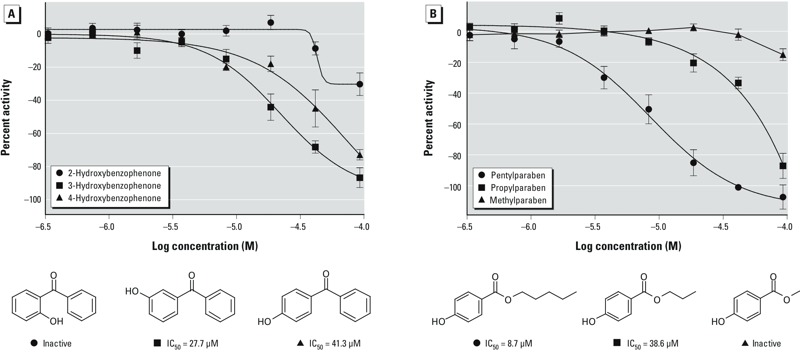
Activity of three hydroxybenzophenone isomers (*A*) and three parabens (*B*). (*A*) Changes in the ability of hydroxybenzophenone isomers to decrease MMP; 3-hydroxybenzophenone was the more potent isomer, and 2-hydroxybenzophenone was inactive in this assay. (*B*) Decreases in MMP for different paraben molecules; activity increased with increasing length of the side chain (and subsequent increase in hydrophobicity).

*Structure–activity relationship (SAR) analysis of compounds that decreased the MMP*. The approximately 8,300 unique compounds within the Tox21 10K compound library were clustered based on their structural similarity using the SOM algorithm (Kohonen 2006), yielding 638 structural clusters and 13 singletons. Of these clusters, 76 (11.7%) were significantly enriched with compounds that decreased the MMP (*p* < 0.05), with 31 of the 76 clusters having a *p*-value lower than 0.001 (Figure 3; see also Supplemental Material, Table S5). The most significantly enriched cluster (Figure 3, row 22, column 19; *p* = 2.04 × 10^–16^; see also Supplemental Material, Table S5) contained 20 of 25 active compounds. The active compounds in this cluster had either an anthraquinone or a benzophenone core with one or more hydroxyl groups. As shown in Figure 4A, 3-hydroxybenzophenone (IC_50_ = 27.7 μM) was slightly more potent than 4-hydroxybenzophenone (IC_50_ = 41.3 μM). The 2-hydroxybenzophenone analog was inactive in the MMP assay. Two other hydroxybenzophenones present in the cluster, 2-hydroxy-4-methoxybenzophenone and mexenone, were not active. Both of these inactive compounds have only one hydroxyl group (in position 2) and one methoxy group (in position 4).

Some structural motifs emerged repeatedly in the clusters enriched for MMP actives. Many of these clusters contained compounds with a phenol moiety, a few had a nitrobenzene core, and three shared a thiazole substructure (Figure 3, red boxes). In the MMP assay, most of the chlorinated organic insecticides present in the library, including toxaphene and aldrin, were active as antagonists. However, chlorendic acid and its anhydride were inactive. Other classes of MMP-active compounds derived from this cluster analysis included parabens, flavonoids, triarylmethane dyes, thiazolidinediones, and ionic liquids, among others (Figure 3; see also Supplemental Material, Table S5). Interestingly, the potencies of parabens seem to correlate with the length of the aliphatic side chain, such as pentylparaben (IC_50_ = 8.7 μM) and propylparaben (IC_50_ = 38.6 μM). Methylparaben, which has a shorter side chain, was inactive in the MMP assay (Figure 4B).

## Discussion

The strategy reported here is a comprehensive utilization of a multiplexed qHTS approach combined with SAR analysis as a first step in the identification of environmental chemicals/drugs and the structural features that decrease MMP. Specifically, we measured changes in MMP using a fluorescent dye (Sakamuru et al. 2012), and changes in intracellular ATP content as a proxy for cytotoxicity using a bioluminescence assay (Crouch et al. 1993) in HepG2 cells in triplicate to profile the Tox21 10K compound library. We used the results of the triplicate runs for each channel [red and green fluorescence, ratio (red/green), cytotoxicity, and compound autofluorescence] to make compound activity calls (see “Activity assignments based on triplicate runs”; see also Supplemental Material, “Reproducibility call,” Tables S2, S3). Having at least three dose–response curves for each compound allowed us to evaluate the technical performance of the assay, thereby increasing our confidence in the compound activity calls. Only 0.55% of the curves had mismatched activities for the MMP assay, and even fewer mismatches were seen with the viability assay (0.03%). The AC_50_ fold change between replicates was generally ≤ 1.5, providing further evidence for high reproducibility in responses. The triplicate data also provided us with the opportunity to evaluate the rationale for the activity assignments based on curve class. High-quality curve classes were more reproducible than low-quality curve classes. This observation provides quantitative evidence supporting the categorization of compounds with high-quality curve classes as active.

More than 11% (913) of the unique compounds in the Tox21 10K library decreased the MMP at 1 hr without apparent cytotoxicity (Figure 2B). An additional 309 compounds significantly decreased both MMP and cell viability (ATP content) over the same concentration range (cytotoxicity is considered excessive when the MMP IC_50_/viability IC_50_ ≤ 3, *p* < 0.05). These compounds were classified as inconclusive antagonists (Figure 2B). The CellTiter Glo® assay is widely used to assess cell viability by measuring intracellular ATP content and is especially well suited, by virtue of its homogeneous assay format and luminescence signal output, to multiplexing with assays generating fluorescence readouts. Assessing cell viability could be useful for differentiating compounds that decreased MMP from those that induced cytotoxicity (and thus resulted in a decrease in the fluorescent signal associated with MMP); in general, cytotoxic compounds were excluded from further data analysis. However, when assessing mitochondrial function, caution must be exercised when interpreting the data from this cell viability assay. Compounds that perturb normal mitochondrial function could cause a decrease in intracellular ATP content. Our data analysis mainly focused on the active antagonist compounds, but the “inconclusive antagonist (cytotoxic)” category of compounds should be further investigated. In fact, rotenone (CASRN 83-79-4) and papaverine (CASRN 61-25-6), two known inhibitors of mitochondrial respiration, and the uncoupler dinitrophenol (CASRN 51-28-5)—and a few of its derivatives—fall into this category. The exact duration of compound treatment prior to application of the MMP detection dye will likely have an effect on the activity assignments mentioned above. On the basis of our previous studies (Attene-Ramos et al. 2013a; Sakamuru et al. 2012), we chose a 1-hr treatment to maximize the detection of compounds that affect mitochondria without triggering cytotoxicity.

Of the 913 active compounds, 79 had an IC_50_ < 1 μM in the MMP assay. The most potent compound in the MMP assay was the PKC modulator bryostatin 1, with an IC_50_ of 9.6 nM. Bryostatin 1 has been reported to induce cell cytotoxicity and/or apoptosis through several mechanisms, including PKC and Bcl-2 modulation as well as alterations in the mitochondria lipid composition that lead to a decrease in the MMP (Cartee et al. 2000; Liu et al. 1999; Wang et al. 2003). Several triarylmethane dyes are among the most potent compounds for this assay, including basic blue 7, methyl violet, and basic red 9. These dyes have been shown to preferentially accumulate in the mitochondria and act as protonophores and/or inhibitors of the electron chain transport resulting in the initiation of apoptosis through the opening of the mitochondrial permeability transition pore (Attene-Ramos et al. 2013a; Kowaltowski et al. 1999). Many organotin compounds, including triethyltin bromide, tributyltin methacrylate, bis(tributyltin)oxide, tributyltin chloride, triphenyltin acetate, and triphenyltin hydroxide, are also among the most potent compounds identified in the present study. Organotins are able to accumulate in biological membranes and interact with many different proteins and lipids (Pagliarani et al. 2013; Saxena 1987). These chemicals are known to inhibit oxidative phosphorylation and induce uncoupling of mitochondrial energy transduction (Aldridge and Street 1964; Sone and Hagihara 1964), causing mitochondrial swelling and opening of the permeability transition pore (Bragadin and Marton 1997; Nishikimi et al. 2001). In the MMP assay, we observed that triorganotins were in general more potent than tetraorganotins or diorganotins and that aryl-substituted organotins were less potent than alkyl substituted ones. This was consistent with previous observations about organotin toxicity (Saxena 1987).

Of the 638 structural clusters in the Tox21 10K compound library, 76 were significantly enriched with compounds that decreased the MMP (*p* < 0.05). Some structural motifs emerged repeatedly within the enriched clusters (Figure 3), and these may constitute mitochondrial toxicophores. The most common motif was a substituted phenol that appeared in many clusters. These compounds with the substituted phenol are known to act as protonophores, facilitating the translocation of protons back into the mitochondrial matrix. The potency and efficacy observed among all the compounds depend, most likely, on the nature of the substituent groups and how they affect the hydroxyl proton acidity and lipophilicity of the entire molecule (Spycher et al. 2008). Interestingly, when we cross-referenced our results with the U.S. EPA’s Chemical Data Reporting database (CDR; http://epa.gov/cdr/, accessed 22 September 2014), the three compounds in the CDR database with the highest use and that were also active in the MMP assay are all substituted phenols [4-aminophenol, CASRN 123-30-8; 4-(1-methyl-1-phenylethyl)phenol, CASRN 599-64-4; and 2,3,4,5,6-pentachlorophenol, CASRN 87-86-5]. Other common structural motifs within active clusters identified by this structural analysis were nitrobenzenes (including nitrophenols) and thiazoles. Nitrophenols are known protonophores and have been used in structural alert models to define uncouplers (Naven et al. 2013).

The most significantly enriched cluster (Figure 3, row 22, column 19; *p* = 2.04 × 10^–16^) contained 20 active compounds (of a total of 25 compounds) that either had an anthraquinone or a benzophenone core with one or more hydroxyl groups. Hydroxyanthraquinones have been reported to be uncouplers of cellular respiration, and their potencies depend on the number and location of the hydroxyl groups (Betina and Kuzela 1987). The effects of benzophenone derivatives on mitochondrial function have not been extensively studied. Our data suggest that the presence of at least one hydroxyl group is necessary (but not sufficient) for the molecule to decrease the MMP. Benzophenone, 4-methylbenzophenone, and 4,4´-dichlorobenzophenone were inactive in the MMP assay. In contrast, most of the hydroxylbenzophenones were active (or inconclusive due to cytotoxicity). In general, compounds with two hydroxyl groups in each ring were more potent than compounds with one or three hydroxyl groups per aromatic ring. The location of the hydroxyl group seems to play an important role in compound potency. Of the monohydroxylated benzophenones, 3-hydroxybenzophenone was more potent than 4-hydroxybenzophenone, and 2-hydroxybenzophenone was inactive in the MMP assay (Figure 4A). This is not true for the dihydroxylated benzophenones, however, where a compound with a hydroxyl in the *ortho* position (2,2´-dihydroxybenzophenone, IC_50_ = 14.1 μM) was active and a compound with a hydroxyl in the *para* position (4,4´-dihydoxybenzophenone) was inconclusive.

The cluster analysis also revealed many categories of compounds capable of decreasing the MMP, including cardiac glycosides, flavonoids, ionic liquids, chlorinated organic insecticides (e.g., toxaphene), parabens, quinolone-based dyes, thiazolidinedione-based drugs (e.g., rosiglitazone), anthracycline-derived antineoplastic drugs (e.g., pirarubicin), and triarylmethane dyes, among others (Figure 3). Some of these structural groups have been previously reported to interact with mitochondria and affect mitochondrial functions. Cardiac glycosides such as ouabain and proscillaridin were among the most potent compounds in the screen. These compounds affect mitochondrial energy production, in part, by decreasing mitochondrial calcium concentration (Liu et al. 2010). Flavonoids have been previously reported to decrease MMP and induce ROS-mediated apoptosis in a variety of cell types, but the specific mechanism is still unknown (Attene-Ramos et al. 2013a; Wang et al. 1999). Triarylmethane dyes accumulate in the mitochondria and inhibit cellular respiration, which may lead to mitochondrial swelling and apoptosis (Kowaltowski et al. 1999). Rosiglitazone and troglitazone are antidiabetic thiazolidinedione drugs that exert their therapeutic effects by activating peroxisome proliferator-activated receptors (PPARs). These drugs have been linked to liver toxicity, in part, by disrupting MMP, which in turn leads to opening of the mitochondrial permeability transition pore and eventually to cell death (Tirmenstein et al. 2002). Parabens, esters of *para*-hydroxybenzoic acid, are used as preservatives in many industries and they have been linked to mitochondrial mediated cytotoxic effects (Nakagawa and Moldeus 1998). The cytotoxic effect of a compound is related to the length of the aliphatic side chain that increases the hydrophobicity of the compound. A similar effect is also observed in the MMP assay, where compounds with longer side chains, such as dodecylparaben (IC_50_ = 4.2 μM) and pentylparaben (IC_50_ = 8.7 μM), are more potent than those with shorter side chains, such as propylparaben (IC_50_ = 38.6 μM) and methylparaben (inactive) (Figure 4B). Finally, four of five anthracycline-based chemotherapy drugs decreased the MMP. Pirarubicin (IC_50_ = 0.6 μM) and valrubicin (IC_50_ = 1.9 μM) were the most potent compounds in this group; aclarubicin hydrochloride, plicamycin, and the structurally related chromomycin A3 were also active, whereas adriamycin hydrochloride was inactive. Anthracyclines are considered to cause cardiotoxicity, at least in part, by affecting mitochondrial function and generating oxidative stress, although the molecular mechanisms is not well understood (Mordente et al. 2012).

## Conclusions

A fundamental objective of the Tox21 collaboration is the generation of a robust, reliable data set in order to prioritize chemicals for more in-depth but lower throughput toxicological studies and to generate reliable computational models. Using a multiplexed qHTS-based assay strategy, we profiled > 8,300 environmental and druglike compounds for their ability to change MMP in HepG2 cells. Monitoring changes in MMP is a suitable first step to screen for mitochondrial dysfunction because compounds that directly or indirectly disrupt a diverse set of mitochondrial and cellular events can potentially affect MMP. The combination of testing each compound as three position-varied 15-point dilution series and including 88 duplicate controls within each plate resulted in a robust screening platform, which in turn allowed us to identify > 800 compounds that decreased the MMP in HepG2 cells. Furthermore, we defined several chemical structural motifs that were frequently associated with a decrease in MMP that could represent putative toxicophores for mitochondrial toxicity. The information generated here may be used to construct computational models to predict new chemical structures capable of disrupting MMP and to generate a tiered approach for selecting compounds for more costly, lower throughput mechanistic studies (e.g., changes in oxygen consumption and inhibition of protein complexes in the respiratory chain). We expect the interpretation of the information obtained using these additional approaches to lead to the prioritization of compounds for *in vivo* testing, providing enhanced understanding of the toxicokinetic and toxicodynamic properties of the compounds, including organ-specific toxicity.

## Supplemental Material

(1.9 MB) PDF
