# Supplementary material for: Profiling of the Tox21 Chemical Collection for Mitochondrial Function to Identify Compounds that Acutely Decrease Mitochondrial Membrane Potential
Source: Environ Health Perspect. 2014 Oct 10;123(1):49–56. doi: 10.1289/ehp.1408642 (PMC4286281; doi:10.1289/ehp.1408642)
Supplement: (1.9 MB) PDF [file ehp.1408642.s001.508.pdf]

Environmental Health Perspectives doi:10.1289/ehp.1408642

Supplemental Material

Profiling of the Tox21 Chemical Collection for Mitochondrial Function to Identify Compounds that Acutely Decrease Mitochondrial Membrane Potential

Matias S. Attene-Ramos, Ruili Huang, Sam Michael, Kristine L. Witt, Ann Richard, Raymond R. Tice, Anton Simeonov, Christopher P. Austin, and Menghang Xia

Cell culture

Human HepG2 cells were cultured in Minimum Essential (Eagle) Medium (ATCC) supplemented with 10% fetal bovine serum (FBS) (Hyclone Laboratories, Logan, UT, USA) and 50 U/mL penicillin and 50 μg/mL streptomycin (Invitrogen, Carlsbad, CA, USA). The cells were maintained at 37°C under a humidified atmosphere and 5% CO_2.

Compound library

The Tox21 10K compound solution testing library was constructed from 20mM stock solution obtained from each of the main Tox21 government partners (EPA, NTP, NCATS). Final 1536 well micro titer Tox21 plates included more than 2000 separately sourced and plated compound replicates, as well as a common set of 88 solutions randomly plated in duplicate across each of the final nine 1536 well Tox21 plates constituting the full library. The list of unique compound substances, including chemical names, Chemical Abstracts Service Registry Numbers (CASRN), and substance description, along with curated chemical structures and auto-generated structure identifiers (formula, systematic names, SMILES, desalted SMILES, InChI) can be downloaded at the EPA DSSTox website at http://www.epa.gov/ncct/dsstox/sdf_tox21s.html (accessed 22 September 2014). Final curve class hit calls are aggregated to a stock solution "Tox21_ID", and qHTS assay results for each Tox21 assay are deposited in PubChem (http://www.ncbi.nlm.nih.gov/pcassay; search term "tox21", accessed 22 September 2014) linked to Tox21_ID and DSSTox unique compound and structure identifiers (http://www.ncbi.nlm.nih.gov/pcsubstance; search term "tox21", accessed 22 September 2014).

Quantitative high throughput screening (qHTS) of mitochondrial membrane potential and cell viability multiplex assay

HepG2 cells were dispensed at 2000 cells/4 µL/well in tissue culture treated 1536-well black wall/clear bottom assay plates (Greiner Bio-One North America, Monroe, NC, USA) using a Multidrop Combi Reagent Dispenser (Thermo Fisher, Waltham, MA, USA). Following incubation of the assay plates at 37°C overnight under a humidified atmosphere and 5% CO_2 in the robotic system incubator (Thermo Fisher), 23 nL of compound or DMSO was transferred into assay plates using a pin tool (Wako, Richmond, VA, USA). All of the compounds were screened at 15 concentrations ranging from 1.18 nM to 92.2 µM. The final concentration of DMSO in the assay was 0.45%. After treatment for 1 hr, 4 µL of 2X m-MPI reagent (1/500 dilution of Mito-MPS solution in assay buffer) was added into the wells using a Flying Reagent Dispenser (FRD) (Aurora Discovery, San Diego, CA, USA) and the plates were incubated for an additional 30 min at 37°C. Fluorescence intensities (485 nm excitation/535 nm emission for green fluorescent monomers; 540 nm excitation/590 nm emission for red fluorescent aggregates) were measured using an Envision plate reader (PerkinElmer; Shelton, CT, USA) (Attene-Ramos et al. 2013; Sakamuru et al. 2012). Data were expressed as the ratio of 590 nm/535 nm, an indicator of MMP. Immediately after, 2 µL of CellTiter-Glo® reagent was added, plates were incubated at room temperature for 30 min, and the luminescence intensity of each well was determined using a ViewLux plate reader (PerkinElmer).

qHTS data analysis

Briefly, raw plate reads for each titration point were first normalized relative to the positive control compound (0%) and DMSO-only wells (-100%) as follows: % Activity = [($V_{compound}$ –

V_{DMSO})/(V_{pos} – V_{DMSO})] x 100, where $V_{compound}$ denotes the compound well value, V_{pos} denotes the median value of the positive control wells, and V_{DMSO} denotes the median values of the DMSO-only wells, and then corrected by applying a NCGC in-house pattern correction algorithm (Southall et al. 2009) using compound-free control plates (i.e., DMSO-only plates) at the beginning and end of the compound plate stack. Concentration–response titration points for each compound were fitted to a four-parameter Hill equation (Hill 1910) yielding concentrations of half-maximal activity (AC50) and maximal response (efficacy) values. Compounds were designated as Class 1–4 according to the type of concentration–response curve observed (Huang et al. 2011; Inglese et al. 2006). Curve classes are heuristic measures of data confidence, classifying concentration–responses on the basis of efficacy, the number of data points observed above background activity, and the quality of fit. The curve sign describe the type of response. Inhibitory curves described compounds that decreased the MMP (antagonist) and active curves were associated with compounds that increase the MMP (agonist) (Huang et al. 2011). All concentration response data and final activity calls are publicly available through PubChem (http://www.ncbi.nlm.nih.gov/pcassay, accessed 22 September 2014) (Assay IDs: 720637, 720635, 720634).

Reproducibility call

Substances were first assigned an activity outcome based on their curve class. Activity outcomes: inactive (class 4), active agonist/antagonist (class 1.1, 2.1), agonist/antagonist (class 1.2, 2.2), inconclusive agonist/antagonist (all other cases). Each activity outcome category was then assigned a score. Activity outcome scores: Active agonist (3), agonist (2), inconclusive agonist (1), active antagonist (-3), antagonist (-2), inconclusive antagonist (-1), inactive (0). The pair-

wise activity outcome score differences for all replicates of each substance were then averaged and the % of inactive calls for the substance calculated to determine the final reproducibility call of the substance. Average pair-wise score difference: active match (<1.1, %inactive call <25%), inactive match (<1.1, %inactive call >50%), mismatch (>2.5), inconclusive (all other cases).

References

Attene-Ramos MS, Huang R, Sakamuru S, Witt KL, Beeson GC, Shou L, et al. 2013a. Systematic study of mitochondrial toxicity of environmental chemicals using quantitative high throughput screening. Chem Res Toxicol 26(9):1323–1332.

Hill AV. 1910. The possible effects of the aggregation of the molecules of haemoglobin on its dissociation curves. J Physiol (London) 40: 4-7.

Huang R, Xia M, Cho MH, Sakamuru S, Shinn P, Houck KA, et al. 2011. Chemical genomics profiling of environmental chemical modulation of human nuclear receptors. Environ Health Perspect 119(8): 1142-1148.

Inglese J, Auld DS, Jadhav A, Johnson RL, Simeonov A, Yasgar A, et al. 2006. Quantitative high-throughput screening: a titration-based approach that efficiently identifies biological activities in large chemical libraries. Proc Natl Acad Sci U S A 103(31): 11473-11478.

Sakamuru S, Li X, Attene-Ramos MS, Huang R, Lu J, Shou L, et al. 2012. Application of a homogenous membrane potential assay to assess mitochondrial function. Physiol Genomics 44(9):495–503.

Southall NT, Jadhav A, Huang R, Nguyen T, Wang Y. 2009. Enabling the Large-Scale Analysis of Quantitative High-Throughput Screening Data. In: Handbook of Drug Screening, Part Second (Seethala R, Zhang L, eds). London:Informa healthcare, 442-463.

Table S1. Screening protocol for the MMP assay.

Step	Parameter	Value	Description
1	Plate cells	cells/4 µL	Plate cells in black clear bottom 1536 well plates, using 8 tip dispense (Multidrop)
2	Incubation time	Overnight	Incubate at 37º C, 5% CO_2
3	Compound addition	23 nL	Pintool transfer of control (1-4 columns) and compound library (5-48 columns).
4	Incubation time	1 hr	Incubate at 37º C, 5% CO_2.
5	Reagent	4 µL	Addition of MMP dye solution (Either Bioraptr or Multidrop)
6	Incubation time	30 min	Incubate at 37º C, 5% CO_2
7	Readout	Envision	Bottom read at Ex: FITC 485; Em: FITC 535
8	Reagent	2 µL	Addition of CellTiter Glo® solution (Either Bioraptr or Multidrop)
9	Incubation time	30 min	Room temperature
10	Readout	ViewLux	Luminescence

Table S2. Compound single channel activity outcome assignments based on curve rank and reproducibility.

Curve rank	Reproducibility call	Activity outcome
>-1 and <1	inactive match	inactive
>-1 and <1	other	inconclusive
>=1	mismatch	inconclusive agonist
>=1	active match	active agonist
>4	other	active agonist
>=1 and <=4	other	inconclusive agonist
<=-1	mismatch	inconclusive antagonist
<=-1	active match	active antagonist
<-4	other	active antagonist
>=-4 and <=-1	other	inconclusive antagonist

Table S3. Compound final assay activity outcome assignments based on multi-channel readouts.

Ratio outcome	Rhodamine outcome	Cell viability outcome	Other conditions	Activity outcome
Inactive	N/A	N/A	N/A	inactive
Inconclusive	N/A	N/A	N/A	inconclusive
Active agonist	agonist	inactive or agonist	N/A	active agonist
Active agonist	agonist	antagonist	$AC_{50\ MMP}/AC_{50\ viability} > 6$	active agonist
Inconclusive agonist	agonist	N/A	N/A	inconclusive agonist
Agonist	agonist	antagonist	$AC_{50\ MMP}/AC_{50\ viability} \leq 6$	inconclusive agonist (cytotoxic)
Active antagonist	antagonist	inactive or agonist	N/A	active antagonist
Active antagonist	antagonist	antagonist	$AC_{50\ MMP}/AC_{50\ viability} > 6$	active antagonist
Inconclusive antagonist	antagonist	N/A	N/A	inconclusive antagonist
Antagonist	antagonist	antagonist	$AC_{50\ MMP}/AC_{50\ viability} \leq 6$	inconclusive antagonist (cytotoxic)
Other	other	other	other	inconclusive

$AC_{50\ MMP}$: Concentrations of half-maximal activity in the MMP assay; $AC_{50\ viability}$: Concentrations of half-maximal activity in the cell viability assay.

Table S4. The 20 most potent active compounds for the MMP screen.

Compound	CASRN	Structure	Potency (nM)	Efficacy (%)
Bryostatin 1	83314-01-6	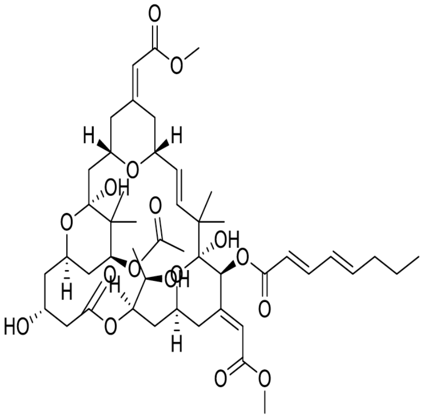	9.58	104.6
Carbocyanine	605-91-4	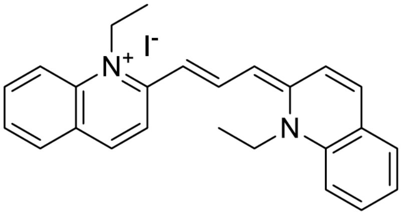	14.7	90.7
Basic blue 7	2390-60-5	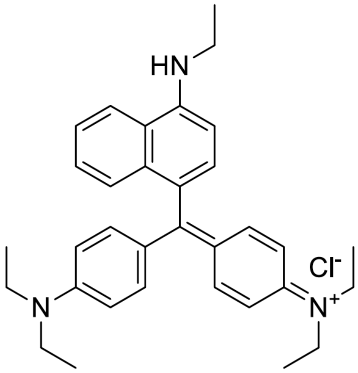	18.5	86.6
Triethyltin bromide	2767-54-6	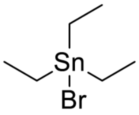	21.7	97.9
Niclosamide	50-65-7	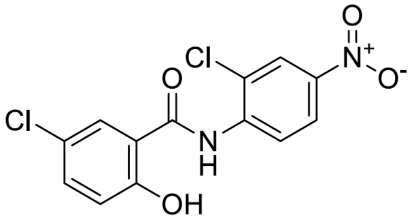	26.3	97.7

Compound	CASRN	Structure	Potency (nM)	Efficacy (%)
1,4-Diaminoanthra-quinone	128-95-0	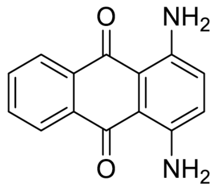	33.1	82.0
2-Hydrazino-4-(4-aminophenyl) thiazole	26049-71-8	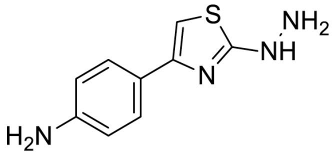	63.5	82.5
Methyl violet	8004-87-3	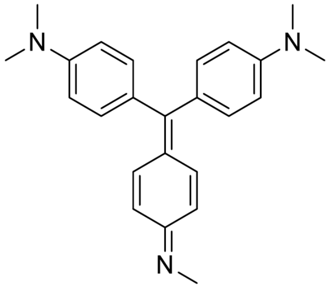	65.3	121.5
Tributyltin methacrylate	2155-70-6	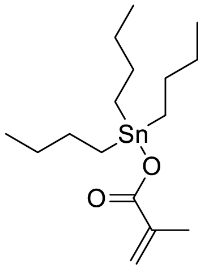	68.6	96.0
1,8-Dihydroxy-4,5-dinitroanthraquinone	81-55-0	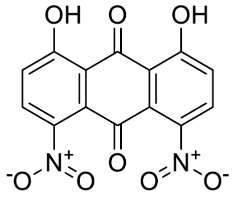	73.4	86.4
Chlorfenapyr	122453-73-0	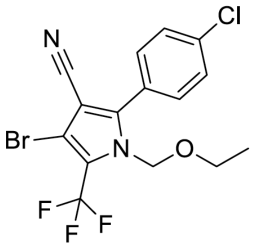	74.1	104.6

Compound	CASRN	Structure	Potency (nM)	Efficacy (%)
C.I. Basic red 9 monohydrochloride	569-61-9	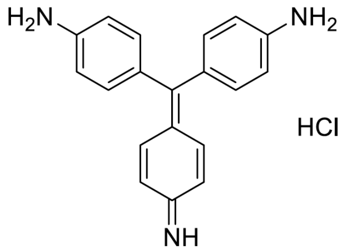	77	95.3
Gentian violet	548-62-9	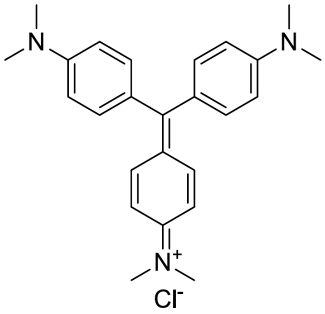	78.8	91.9
Proscillaridin	466-06-8	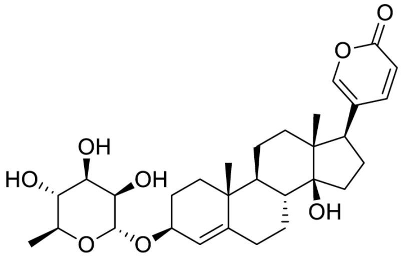	86.8	72.4
Bis(tributyltin)oxide	56-35-9	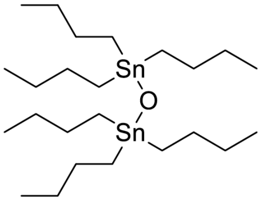	91.1	112.1
Dithiazanine iodide	514-73-8	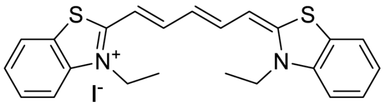	95.8	85.4
Fluorescein	2321-07-5	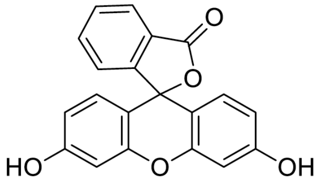	105.3	112.5

Compound	CASRN	Structure	Potency (nM)	Efficacy (%)
Tributyltin chloride	1461-22-9	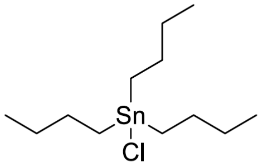	106.7	96.9
Triphenyltin acetate	900-95-8	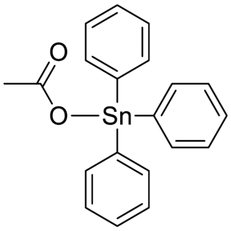	117.4	98.7
Triphenyltin hydroxide	76-87-9	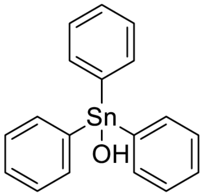	122	94.7

Table S5. A list of active antagonist clusters with representative scaffolds.

Cluster	Number of actives	Number of compounds in the cluster	Log p	Representative scaffold
**22.19**	**20**	**25**	**15.7**	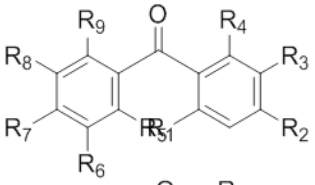 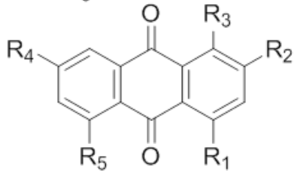
**24.19**	**21**	**28**	**13.9**	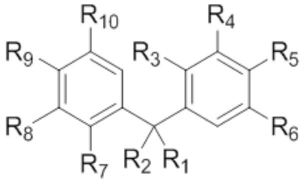
**26.15**	**18**	**25**	**11.5**	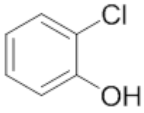 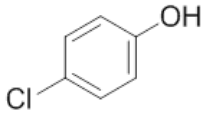
**15.17**	**13**	**16**	**9.5**	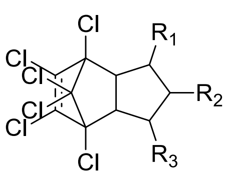 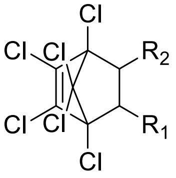
**12.19**	**13**	**22**	**9.1**	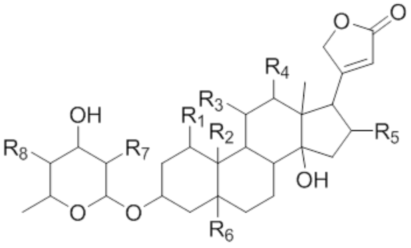
**24.18**	**10**	**15**	**8.7**	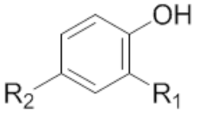

Cluster	Number of actives	Number of compounds in the cluster	Log p	Representative scaffold
25.18	11	20	7.6	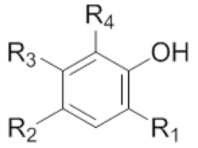
25.19	12	20	7.6	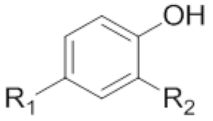
23.17	5	8	7.5	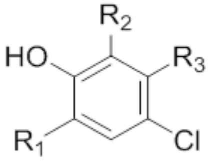
20.1	14	27	7.4	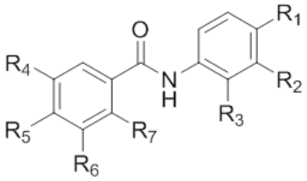
21.6	6	9	5.1	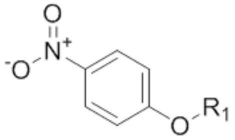
21.5	6	12	5	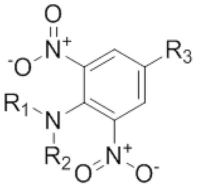
34.15	8	12	5	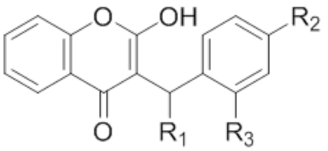
22.6	5	5	4.7	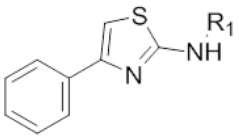

Cluster	Number of actives	Number of compounds in the cluster	Log p	Representative scaffold
33.18	8	17	4.4	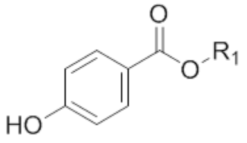
19.5	8	25	4.3	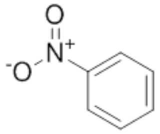
11.12	6	8	4.3	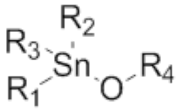
33.15	8	18	4.2	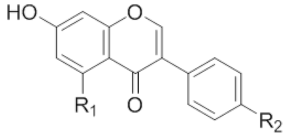 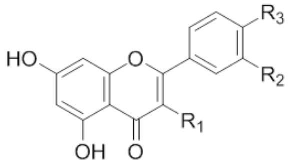
19.10	5	9	3.8	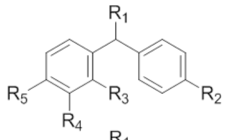 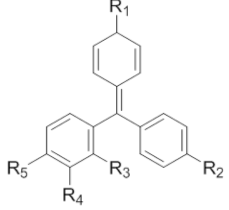
24.16	5	9	3.8	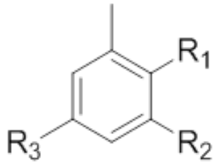

Cluster	Number of actives	Number of compounds in the cluster	Log p	Representative scaffold
23.7	9	24	3.7	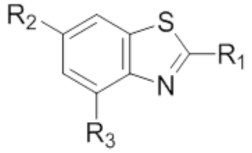
10.5	9	21	3.5	
28.17	6	10	3.5	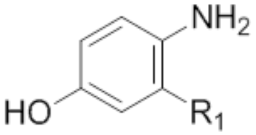
35.17	8	18	3.3	
17.5	6	11	3.2	
23.18	6	11	3.2	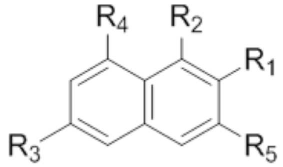
22.16	7	15	3.1	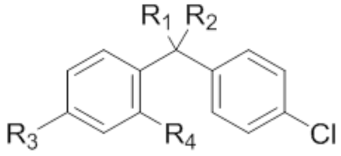
21.7	3	5	3.1	
29.9	3	5	3.1	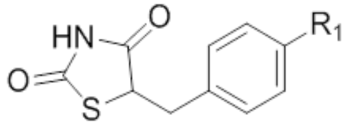
32.5	4	5	3.1	
1.6	12	38	3.1	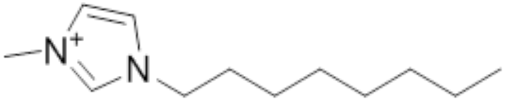

Log p: logarithm of P value.

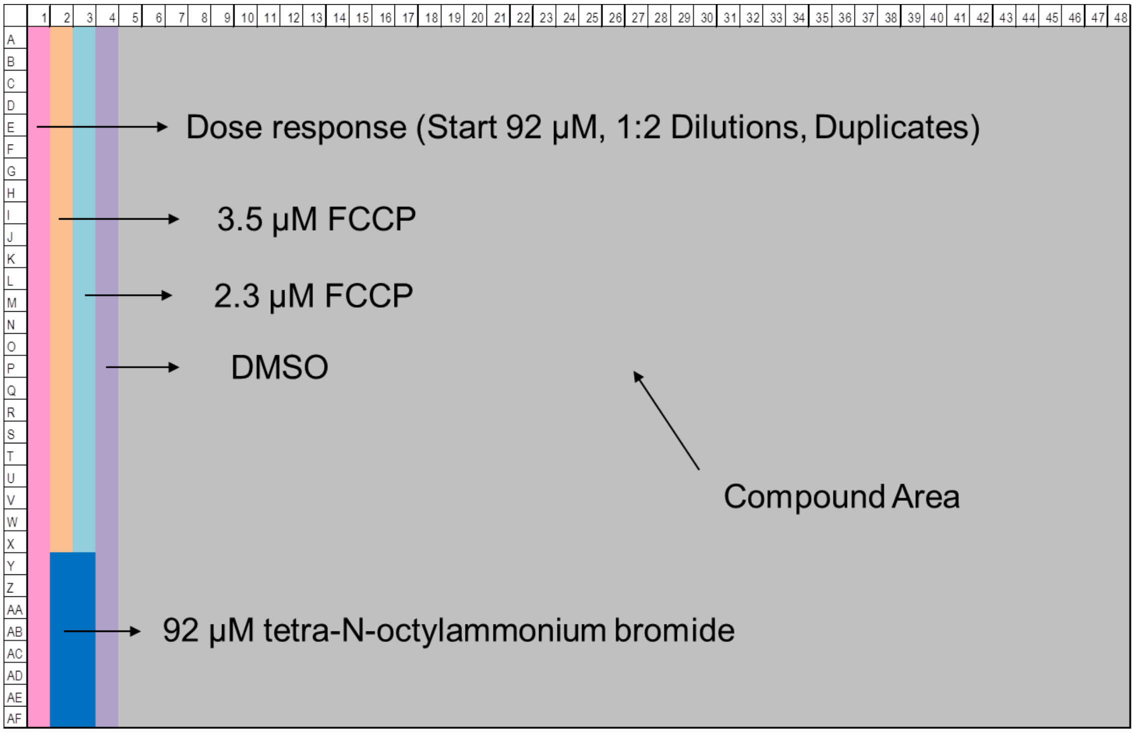

Figure S1. qHTS MMP assay plate map showing the location of the controls used in the screen. Column 1, Carbonyl cyanide 4-(trifluoromethoxy) phenylhydrazone (FCCP) titration ranging from 2.8 nM to 92 µM in duplicate; top 24 wells of column 2, 3.5 µM FCCP and column 3, 2.3 µM FCCP; bottom 8 wells from columns 2 and 3, 92 µM tetra-N-octylammonium bromide, the positive control for the cell viability assay; and column 4, Dimethyl sulfoxide (DMSO) only. Columns 5 to 48 are the compound areas transferred from the compound library plates.

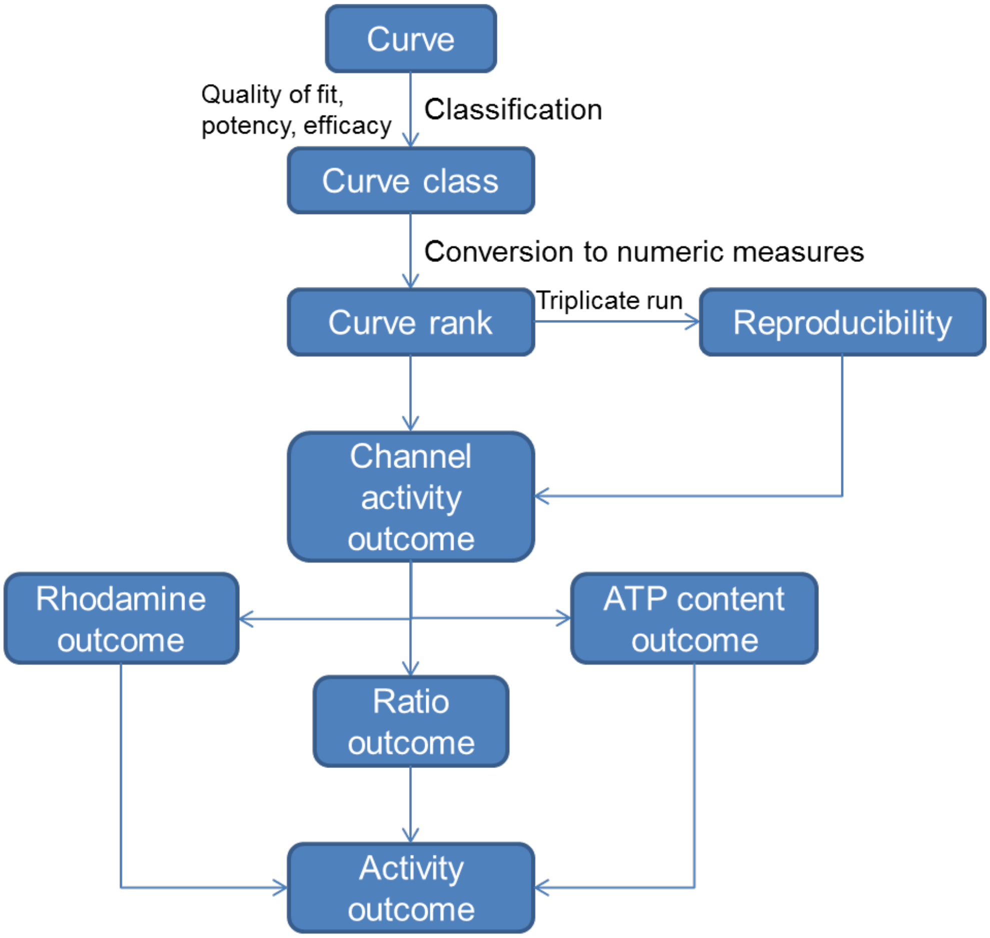

Figure S2. Schematic overview of activity assignment process.

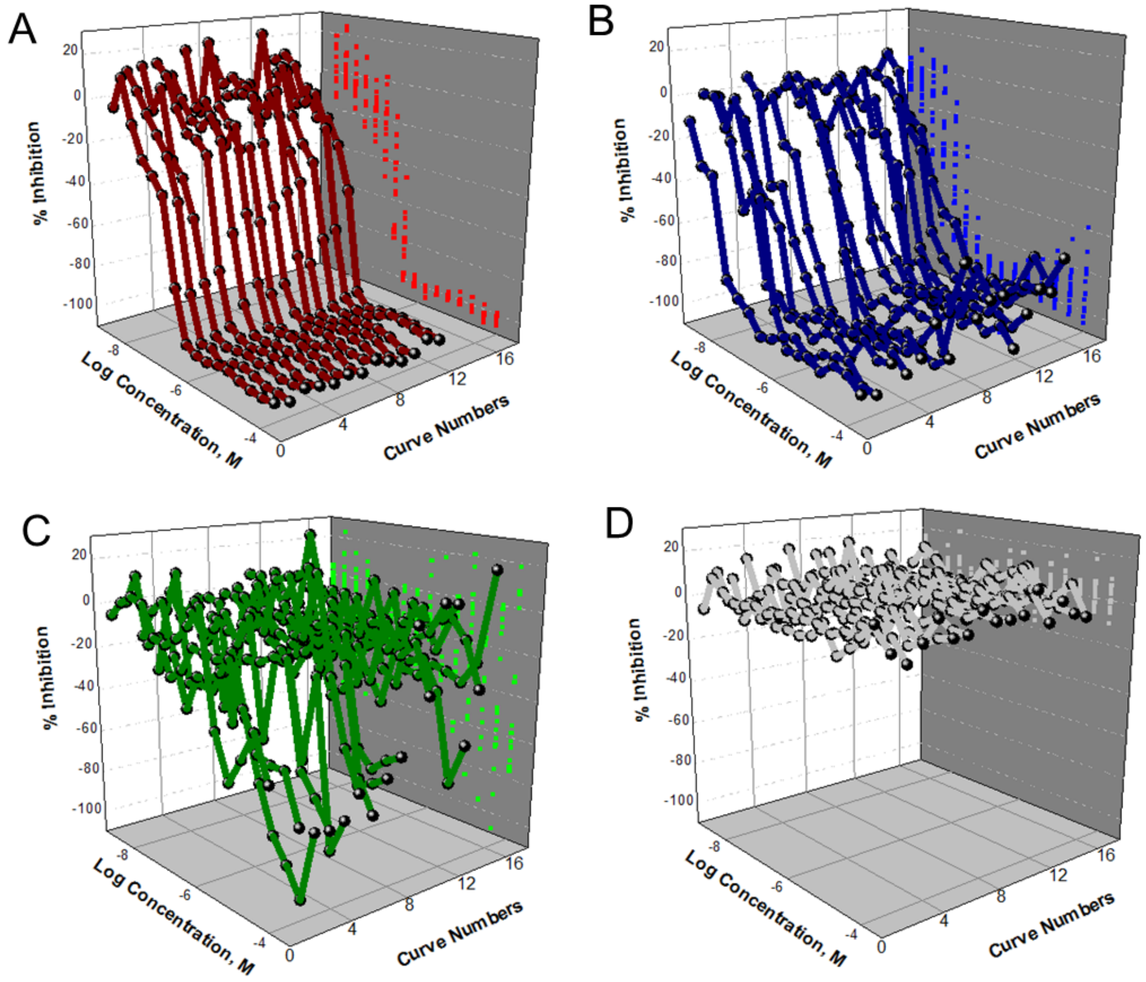

Figure S3. Representative qHTS concentration response curves: (A) Curves for carbonyl cyanide 4-(trifluoromethoxy) phenylhydrazone (FCCP, positive control); (B) Curves for curve classes 1.1, 1.2, or 2.1 compounds; (C) Curves for curve classes 1.3, 1.4, 2.2, 2.3, 2.4, and 3 compounds; (D) Inactive compounds (curve class 4).
